# Supplementary material for: Comprehensive benchmarking of CITE-seq versus DOGMA-seq single cell multimodal omics
Source: Genome Biol. 2022 Jun 23;23:135. doi: 10.1186/s13059-022-02698-8 (PMC9219143; doi:10.1186/s13059-022-02698-8)
Supplement: Supplementary file 1 — Additional file 1: Figures S1-S4. Figure S1. Additional comparison between DIG and LLL conditions. Figure S2. Additional comparison between CITE-seq and DOGMA-seq, part 1. Figure S3. Additional comparison between CITE-seq and DOGMA-seq, part 2. Figure S4. Alternative comparisons of transcript measurements where CITE-seq and DOGMA-seq RNA libraries were aligned to both exons and introns. [file 13059_2022_2698_MOESM1_ESM.pdf]

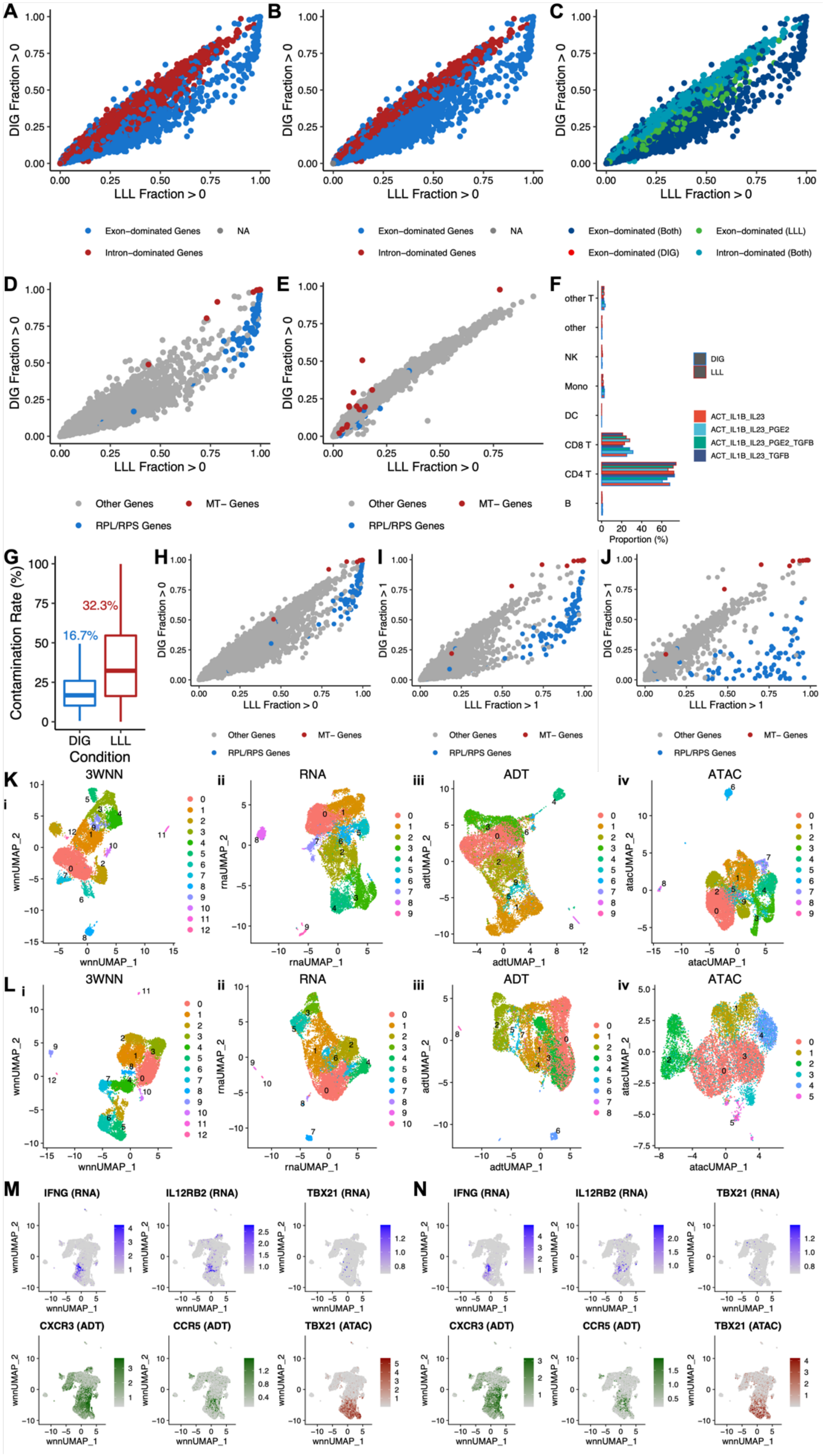

A
B
C
DIG Fraction > 0
LLL Fraction > 0
Exon−dominated Genes
NA
Intron−dominated Genes
Exon−dominated (Both)
Exon−dominated (LLL)
Exon−dominated (DIG)
Intron−dominated (Both)
D
E
F
Other Genes
MT− Genes
RPL/RPS Genes
other T
other
NK
Mono
DC
CD8 T
CD4 T
B
Proportion (%)
DIG
LLL
ACT_IL1B_IL23
ACT_IL1B_IL23_PGE2
ACT_IL1B_IL23_PGE2_TGFB
ACT_IL1B_IL23_TGFB
G
Contamination Rate (%)
32.3%
16.7%
DIG
LLL
Condition
H
I
J
DIG Fraction > 1
LLL Fraction > 1
K
L
i
ii
iii
iv
3WNN
RNA
ADT
ATAC
wnnUMAP_1
wnnUMAP_2
rnaUMAP_1
rnaUMAP_2
adtUMAP_1
adtUMAP_2
atacUMAP_1
atacUMAP_2
M
N
IFNG (RNA)
IL12RB2 (RNA)
TBX21 (RNA)
CXCR3 (ADT)
CCR5 (ADT)
TBX21 (ATAC)

Fig. S1 | Additional comparison between DIG and LLL conditions

(A-C) Pairwise comparison of gene detection frequencies under LLL (x-axis) and DIG (y-axis) conditions. Each point represents a single gene. Blue points highlight exon-dominated genes; red points highlight intron-dominated genes. NA means proportion of exonic UMIs is not available. (A) An exon-dominated gene is defined as a gene with proportion of exonic UMIs (DIG) > 0.5. An intron-dominated gene is defined as a gene with proportion of exonic UMIs (DIG) ≤ 0.5. (B) An exon-dominated gene is defined as a gene with proportion of exonic UMIs (LLL) > 0.5. An intron-dominated gene is defined as a gene with proportion of exonic UMIs (LLL) ≤ 0.5.

(C) Genes defined as exon-dominated genes under both DIG and LLL conditions are highlighted in blue; genes defined as exon-dominated genes only under DIG condition are highlighted in red; genes defined as exon-dominated genes only under LLL condition are highlighted in green; genes defined as intron-dominated genes under both DIG and LLL conditions are highlighted in cyan.

(D-E) Pairwise comparison of gene detection frequencies under LLL (x-axis) and DIG (y-axis) conditions. Each point represents a single gene. Blue points highlight ribosomal protein genes (RPL/S); red points highlight mitochondrial genes (MT-). Grey points are all other genes. (D) Genes were mapped to exons only under both DIG and LLL conditions. (E) Genes were mapped to introns only under both DIG and LLL conditions.

(F) Bar plot showing proportions of labeled clusters in DOGMA-seq data under DIG and LLL conditions, split by treatment conditions.

(G) Boxplot showing ambient RNA contamination rate per cell (estimated by DecontX based on raw matrices containing empty droplets) between DIG and LLL conditions. Median values are indicated with corresponding colors. Median ratio = 0.52.

(H-J) Pairwise comparison of gene detection frequencies under LLL (x-axis) and DIG (y-axis) conditions. Each point represents a single gene. Blue points highlight ribosomal protein genes (RPL/S); red points highlight mitochondrial genes (MT-). Grey points are all other genes. (H) Gene detection rates, defined as the fraction of cells with UMIs > 0, were calculated based on decontaminated RNA count matrices. (I) Gene detection rates, defined as the fraction of cells with UMIs > 1, were calculated based on original RNA count matrices. (J) Gene detection rates, defined as the fraction of cells with UMIs > 1, were calculated based on decontaminated RNA count matrices.

(K-L)(i) 3WNN UMAP plot showing clusters identified in 3WNN clustering of DOGMA-seq data. (ii) UMAP plot showing clusters identified based on RNA of DOGMA-seq data. (iii) UMAP plot showing clusters identified based on ADT of DOGMA-seq data. (iv) UMAP showing clusters identified based on ATAC of DOGMA-seq data. (K) Under DIG condition, with resolution = 0.2 for all clustering. (L) Under LLL condition, with resolution = 0.2 for all clustering.

(M-N) "Harmonized" 3WNN UMAP plots highlighting canonical markers for Th1 cells in DOGMA-seq data. ATAC marker s motif activity (the deviations in chromatin accessibility across the set of regions related to the motif) calculated from ATAC-seq peaks. (M) Under DIG condition. Cluster 3 in Fig. 1Ni is enriched for Th1 cells based on CXCR3 ADT, CCR5 ADT, and *TBX21* ATAC signals. (N) Under LLL condition. Cluster 2 in Fig. 1Oi is enriched for Th1 cells based on CXCR3 ADT, CCR5 ADT, and *TBX21* ATAC signals.

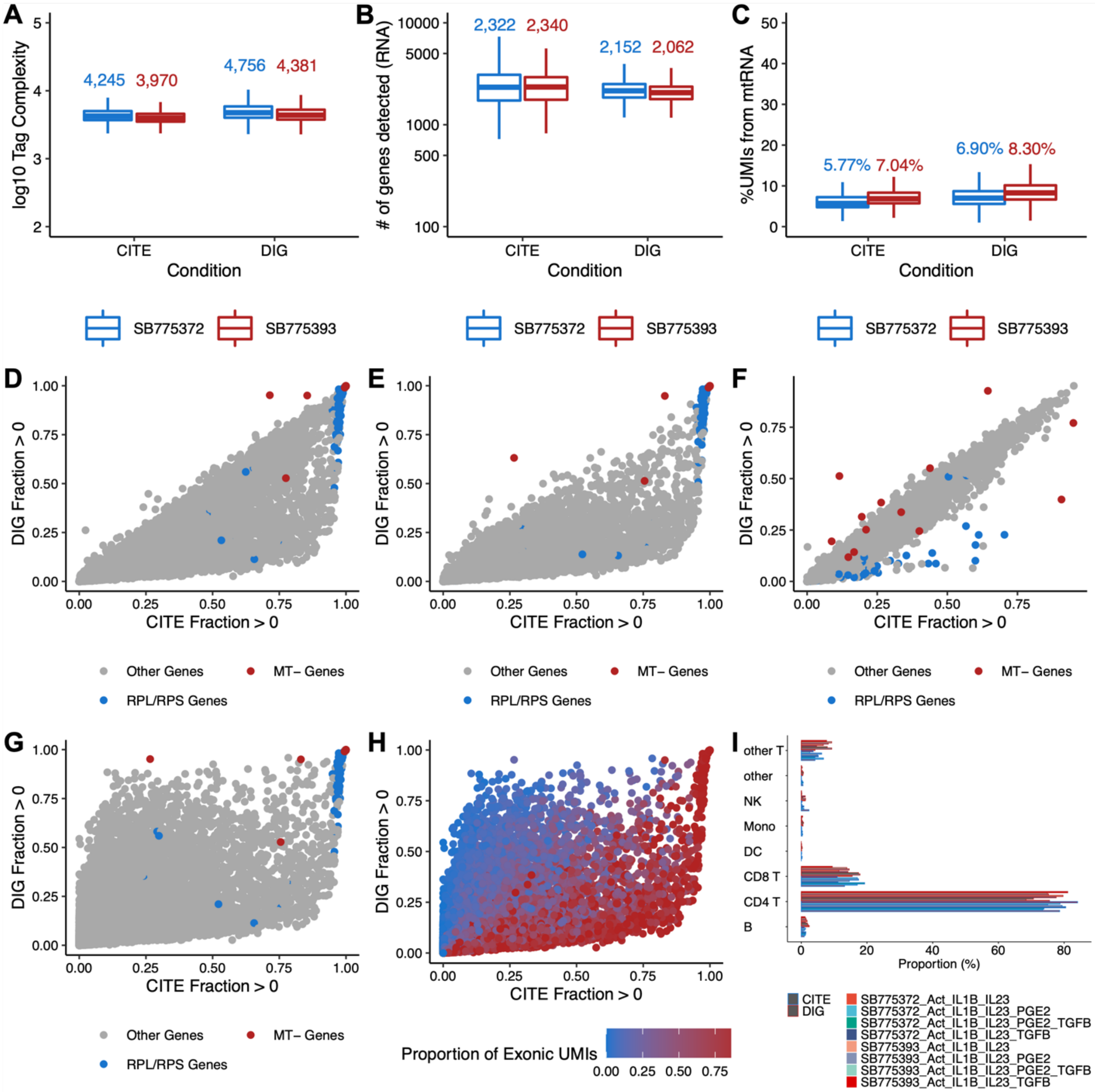

Fig. S2 | Additional comparison between CITE-seq and DOGMA-seq, part 1

(A-C) Boxplot showing quality control metric comparisons between CITE-seq and DOGMA-seq, split by donors (SB775372 or SB775393). Median values are indicated with corresponding colors. (A) Protein tag complexity per cell. Median ratio = 0.89 or 0.91. (B) Number of genes per cell. Median ratio = 1.08 or 1.13. (C) Percent of UMIs mapped to mtRNA per cell. Median ratio = 0.84 or 0.85.

(D-G) Pairwise comparison of gene detection frequencies in CITE-seq (x-axis) and DOGMA-seq (y-axis). Each point represented a single gene. Blue points highlighted ribosomal protein genes (RPL/S); red points highlighted mitochondrial genes (MT-). Grey points are all other genes. (D) Genes were mapped to both exons and introns in both CITE-seq and DOGMA-seq. (E) Genes were mapped to exons only in both CITE-seq and DOGMA-seq. (F) Genes were mapped to

introns only in both CITE-seq and DOGMA-seq. (G) Genes were mapped to exons only in CITE-seq, and mapped to both exons and introns in DOGMA-seq.
(H) Pairwise comparison of gene detection frequencies in CITE-seq (x-axis) and DOGMA-seq (y-axis). Each point represented a single gene. The color scale indicated proportion of exonic UMIs (DOGMA-seq).
(I) Bar plot showing proportions of labeled clusters in DOGMA-seq and CITE-seq data, split by donors and treatment conditions.

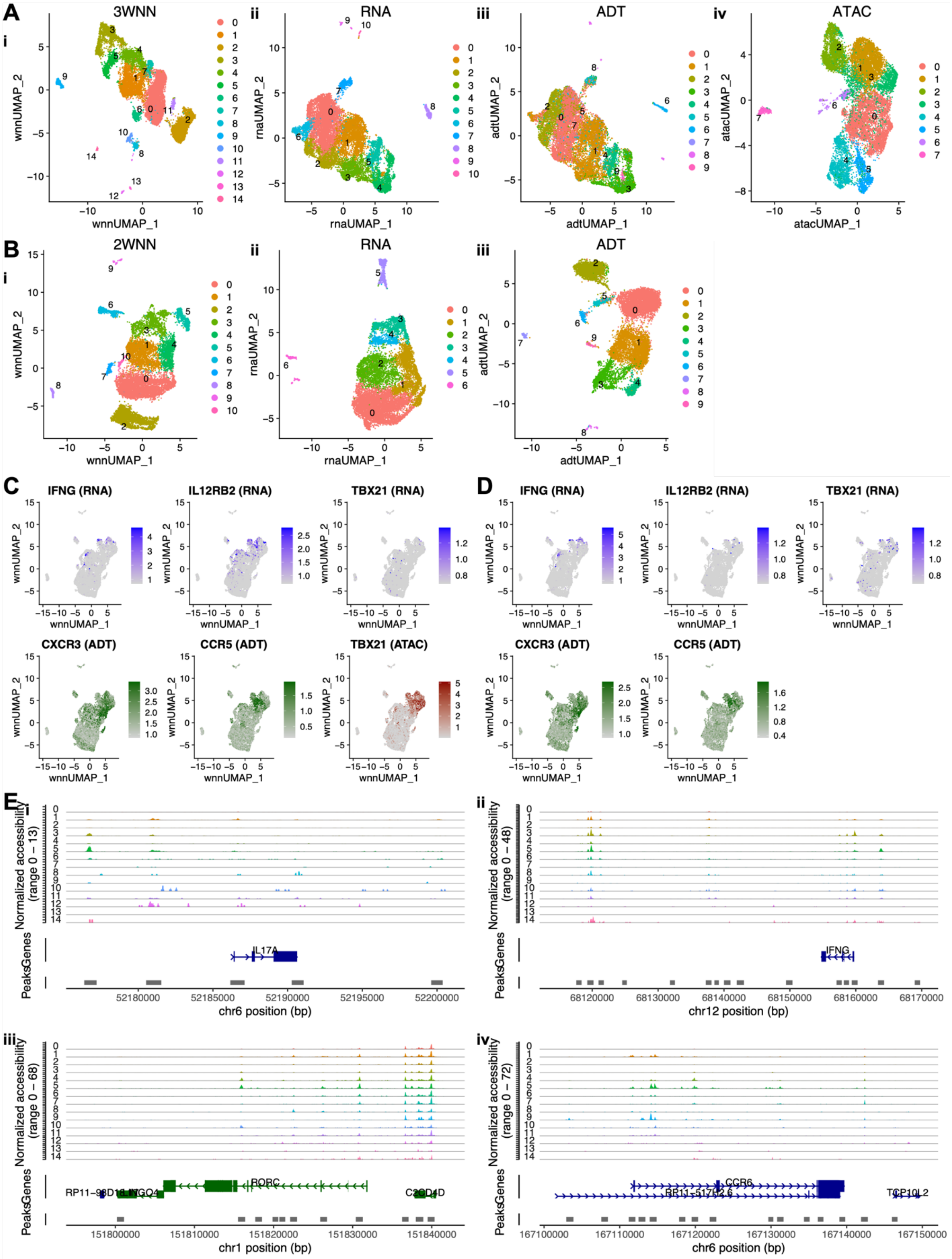

Fig. S3 | Additional comparison between CITE-seq and DOGMA-seq, part 2

(A)(i) 3WNN UMAP plot showing clusters identified in 3WNN clustering of DOGMA-seq data. (ii) UMAP plot based on RNA showing clusters identified in clustering based on RNA of DOGMA-seq data. (iii) UMAP plot based on ADT showing clusters identified in clustering based on ADT of DOGMA-seq data. (iv) UMAP plot based on ATAC showing clusters identified in clustering based on ATAC of DOGMA-seq data. With resolution = 0.2 for all clustering.
(B)(i) 2WNN UMAP plot showing clusters identified in 2WNN clustering of CITE-seq data. (ii) UMAP plot based on RNA showing clusters identified in clustering based on RNA of CITE-seq data. (iii) UMAP plot based on ADT showing clusters identified in clustering based on ADT of CITE-seq data. With resolution = 0.2 for all clustering.
(C) "Harmonized" 2WNN UMAP plots highlighting canonical markers for Th1 cells in DOGMA-seq data. ATAC marker was motif activity (the deviations in chromatin accessibility across the set of regions related to the motif) calculated from ATAC-seq peaks. Cluster 3 in Fig. 2Ii is enriched for Th1 cells based on CXCR3 ADT, CCR5 ADT, and *TBX21* ATAC signals.
(D) "Harmonized" 2WNN UMAP plots highlighting canonical markers for Th1 cells in CITE-seq data.
(E)(i-iv) ATAC-seq peaks in genomic regions around *IL17A, IFNG, RORC, CCR6*, canonical markers for Th17 and Th1 cells. Cluster 5 in Fig. 2Ii is enriched for Th17 cells based on peaks around *IL17A, RORC, and CCR6.* Cluster 3 in Fig. 2Ii is enriched for Th17 cells based on peaks around *IFNG.*

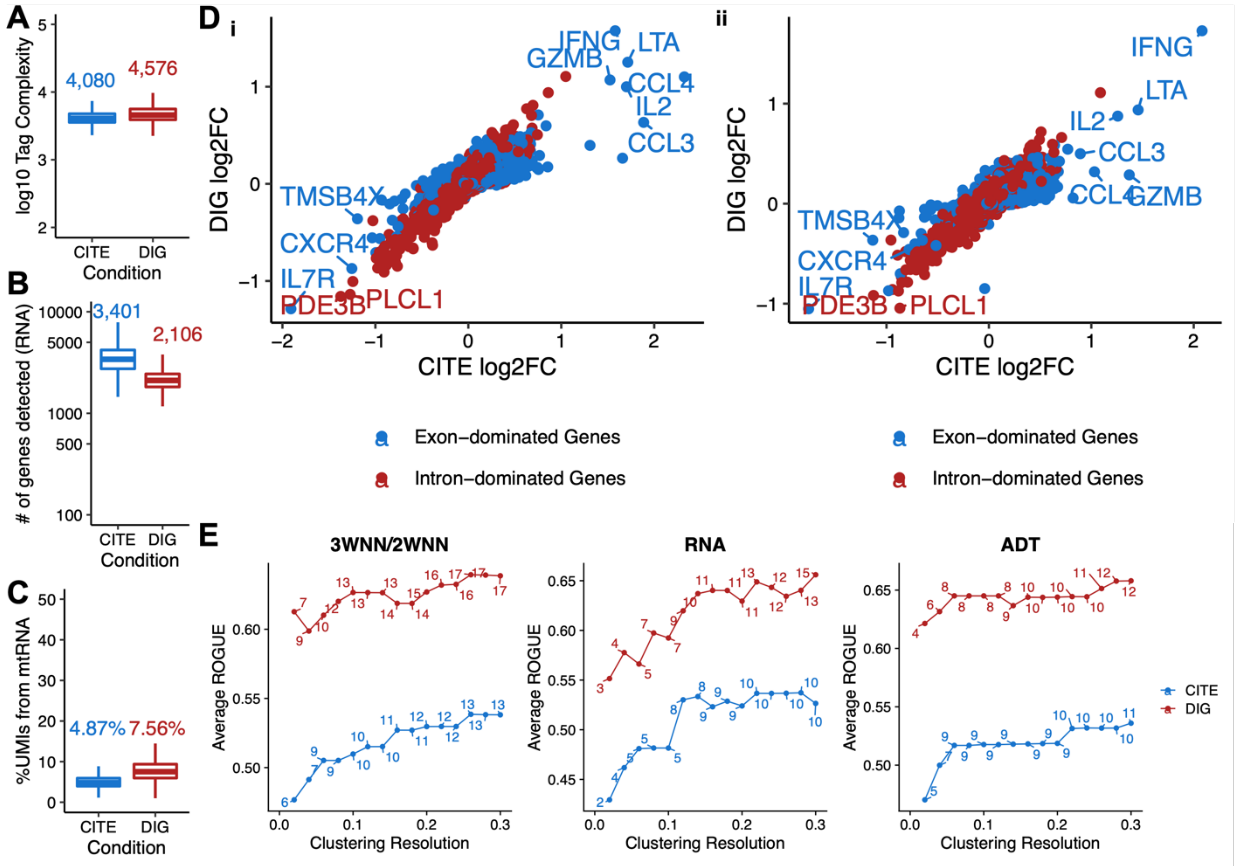

Fig. S4 | Alternative comparisons of transcript measurements where CITE-seq and DOGMA-seq RNA libraries were aligned to both exons and introns

(A-C) Boxplot showing quality control metric comparisons between CITE-seq and DOGMA-seq. Median values are indicated with corresponding colors. (A) Protein tag complexity per cell. Median ratio = 0.89. (B) Number of genes per cell. Median ratio = 1.61. (C) Percentage of UMIs mapped to mtRNA per cell. Median ratio = 0.64.

(D)(i) Correlation of gene fold change ($\log_2$) as detected by CITE-seq and DOGMA-seq in two groups of T cells from donor SB775372 that were both activated and cultured with IL-1β and IL-23, and one of the two groups was also cultured with PGE2. Selected intron-dominated genes are highlighted in red; selected exon-dominated genes are highlighted in blue. (ii) in samples from donor SB775393, as (i).

(E) Line plots showing average ROGUE (purity of identified clusters) for clustering based on 3WNN/2WNN, RNA, and ADT spaces, with resolution ranging from 0.02 to 0.3. Number of clusters identified in each clustering was labeled.
