# Supplementary material for: Comprehensive benchmarking of CITE-seq versus DOGMA-seq single cell multimodal omics
Source: Genome Biol. 2022 Jun 23;23:135. doi: 10.1186/s13059-022-02698-8 (PMC9219143; doi:10.1186/s13059-022-02698-8)
Supplement: Supplementary file 2 — Additional file 2. [file 13059_2022_2698_MOESM2_ESM.docx]

Review History

**First round of review**

**Reviewer 1**

**Were you able to assess all statistics in the manuscript, including the appropriateness of statistical tests used?**

Yes

**Comments to author:**

The manuscript by Xu et al. presents a comparison between two permeabilization approaches (digitonin and paraformaldehyde fixation with low-loss lysis) to DOGMA-seq, a recently proposed method for the simultaneous profiling of transcription, surface protein expression, and chromatin accessibility of individual cells (Mimitou et al. Nat. Biotechnol. 39 (2021)). The authors compare the results of DOGMA-seq to those of CITE-seq on PBMCs. There are several experimental methods for the simultaneous profiling of transcription, surface protein expression, and chromatin accessibility of individual cells that have been recently published (DOGMA-seq, TEA-seq). Optimizing these methods and performing systematic comparisons between them and methods that simultaneously profile two modalities but are well established, such as CITE-seq, SNARE-seq, and commercially available analogues (e.g. by 10x Genomics), would be of interest to the growing community of researchers using these technologies. However, in my opinion the study presented in this manuscript falls short in two important aspects:

1. The scope of the study is too narrow and will likely be of interest to a reduced number of people. DOGMA-seq and TEA-seq have not yet been widely adopted by other researchers, and it is yet unclear if they will be broadly adopted. A systematic comparative study could be useful for potential future adopters of these methods. However, this manuscript focuses on only one of these approaches (DOGMA-seq) and the optimization and conclusions are based on only one tissue (PBMCs). The utility of this study would be increased if both DOGMA-seq and TEA-seq were considered, the conclusions were drawn based on multiple tissues, and other well-established technologies for single-cell multi-omics were considered in the comparisons (e.g. 10x multiome for concurrent scRNA+scATAC).

2. Some of the analyses presented in the manuscript lack rigor and raise doubts about the validity of the conclusions. Specifically:

2a. Comparing the number of clusters obtained with a clustering algorithm (in this case Louvain implemented in Seurat) using fixed parameters is by no means a metric of information content or quality. There are many aspects that affect the number of clusters obtained in Louvain clustering (or any other clustering algorithm) and are not necessarily related to the quality or discreteness of the data. For example, the number of cells, the density of the latent space, the scale and distribution of pairwise distances, etc. A more rigorous approach should be used here. For example, the authors could subsample reads (not UMIs) and cells so that the libraries have the same number of cells and UMIs. Then, they could then consolidate the subsampled libraries in a common latent space (e.g. using Harmony) and cluster this latent space to identify distinct cell populations. They could then perform differential expression analysis for each cell population and compare the number of differentially expressed genes obtained in each cell population by using only cells from one library at a time. Alternatively, they could use notions from information theory to quantify the amount of information content.

2b. Comparing the transcriptome of CITE-seq and DOGMA-seq, when CITE-seq reads are aligned to exons and DOGMA-seq reads are aligned to both exons and introns does not make sense. Only comparisons where reads from both assays are aligned to the same features (Figs. S2D-F) should be considered.

2c. A critical aspect that can negatively affect the quality of the libraries in DOGMA-seq is the presence of mRNA from lysed cells in the buffer. However, none of the metrics included in this study directly account for that. The study would greatly benefit from a human-mouse cell mixture experiment where the amount of background mRNA can be directly assessed in each permeabilization condition and technology by looking at the fraction of mouse UMIs when profiling human cells (and the fraction of human UMIs when profiling mouse cells).

**Reviewer 2**

**Were you able to assess all statistics in the manuscript, including the appropriateness of statistical tests used?**

N/A

**Comments to author:**

Line 48 - spelling of DOGMA
A reference/supplementary figure to the change in lysis time and concentration of DIG for the lysis step would be advantageous. The authors also state that after 1 min incubation in 0.0075% DIG 0.5% of the cells showed signs of permeabilization. Is this what the authors intended to state in the results section?
The results section states that each cell population was then split into the DIG and LLL permeabilization groups, does this mean that all activation/stimulation steps were treated separated or combined together. It is not very clear from the results section.
How is tag complexity measured?
The authors state that whilst differential cell surface protein detection was seen between the two methods, this did not result in different protein tag signals. Could the authors then explain what was the differential measure that could be detected, was it different numbers of UMIs per ADT, dynamic range of signal?
The authors state that they can detect a slightly higher detection rate for some genes after LLL permeabilization (Fig 1L), is there any specific about these genes which have a higher detection level in the LLL procedure?
Does the detection of more RPL/RPS genes actually provide more information/definition to the multimodal analysis?
Could the authors explain why the data had to be batch corrected using Harmony before running the 3WNN? Where all activation/stimulation conditions pooled together for the analysis?
Whilst it is true that an additional cluster can be seen in the UMAP plots for RNA (LLL) and ADT (DIG) do these clusters actually improve the definition of the landscapes? Do the authors know the identity of these cells, are they the same sub-cell type, detected by the different omic methods?
The authors state that they can use the ATAC data to identify specific sub-types of T cells within the data. It would be better to state which markers/genes they are looking at in the text. Also would it not be of advantage to show the same gene/marker in all 3 omics to show the difference in detection, the authors have done this for CCR6 and RORC in two of the omics but not all three.
When showing the ATAC seq data on the wnnUMAP, what exactly are the authors showing, is this all the open regions detectable within the gene highlighted?

The authors do not conclusively prove or conclude at the end of Figure 1 which method is better to define the landscape. Do they benefit from having more information from the ADT, mRNA or ATAC to define the landscape? Is there really a qualitive or quantitative difference between the two methods?

It is not immediately clear from the text that the two donors were kept separately but used for both protocols, this should be made clearer in the schematic in Figure 2 or mentioned in the text.
Is there an explanation as to why the complexity of the ADT library is increased in the second DIG experiment? Is this degree of variability normal between repeats of the process and if this is the case then how can qualitative comparisons between the methods/technical repeats be made?

The protein tag detection rates of DOGMA-seq are clearly lower than those of CITE-Seq and yet the complexity of the library is higher than that of the CITE-Seq experiment. Does this signify that the complexity of the ADT library is not a good metric to determine the "success" of the protocol?
The same markers highlighted in Fig 2Fi-ii are also shown in Fig 1, does this again show that these markers are so upregulated compared to any other surface protein, that they dominate the ADT library?
What is the actual dynamic detection range of all the 163 protein tags in the ADT library when measured by the different protocols?
Does this analysis suggest that whilst the number of genes detected using CiTE-Seq and DOGMA-Seq protocols is similar, the more robust expression is detected using the CITE-Seq protocol?

The authors claim that there are less clusters seen in the RNA and ADT UMAPs of the DOGMA-Seq experiments, due to better quality data in the CITE-Seq experiments. Supplementary figure 3 shows that there are more clusters in S3Aii and iii than in S3Bii and iii, so this statement is inaccurate. The ADT UMAP of the CITE-Seq experiment appears to be much better resolved than that of the DOGMA-Seq but the number of clusters is less in CITE-Seq when compared to DOGMA-Seq.

The predicted celltype labelling on all UMAPs is unsatisfactory, whilst it is understandable that the authors have corroborated the cell type association using Azimuth, the tri-modal approach taken in DOGMA-seq should allow much better celltype prediction as the authors elude to in the text. It would be of benefit to the readers of this article if the authors tried to define the celltypes based on the individual clusters rather than combining clusters and labelling large areas of the UMAPs as CD4/CD8 T cells, this is not particularly informative to the reader.

The authors have also performed several different stimulations of the isolated T cells, where is all of this data from the individual stimulations? It would be of benefit to the general audience of this manuscript to be able to see how these stimulations change the landscape, whilst it is beyond the scope of this manuscript to delve into the differences seen in the epigenetic landscape upon stimulation and how this correlates with gene expression changes or surface protein phenotype it would be reassuring to know that this data was available as a resource for the community.
In the methods section there is mention of using Hastags to separate the donors and condition, this was not mentioned within the manuscript, does this additional layer of multiplexing affect the experimental procedure?
The authors mention that the raw and processed data will be deposited to GEO upon acceptance of the paper, the data needs to be submitted (but kept private) before the paper can be accepted and the same for the analysis code.

**Authors Response**

**Point-by-point responses to the reviewers’ comments:**

Reviewer #1

The manuscript by Xu et al. presents a comparison between two permeabilization approaches (digitonin and paraformaldehyde fixation with low-loss lysis) to DOGMA-seq, a recently proposed method for the simultaneous profiling of transcription, surface protein expression, and chromatin accessibility of individual cells (Mimitou et al. Nat. Biotechnol. 39 (2021)). The authors compare the results of DOGMA-seq to those of CITE-seq on PBMCs. There are several experimental methods for the simultaneous profiling of transcription, surface protein expression, and chromatin accessibility of individual cells that have been recently published (DOGMA-seq, TEA-seq). Optimizing these methods and performing systematic comparisons between them and methods that simultaneously profile two modalities but are well established, such as CITE-seq, SNARE-seq, and commercially available analogues (e.g. by 10x Genomics), would be of interest to the growing community of researchers using these technologies. However, in my opinion the study presented in this manuscript falls short in two important aspects:

1. The scope of the study is too narrow and will likely be of interest to a reduced number of people. DOGMA-seq and TEA-seq have not yet been widely adopted by other researchers, and it is yet unclear if they will be broadly adopted. A systematic comparative study could be useful for potential future adopters of these methods. However, this manuscript focuses on only one of these approaches (DOGMA-seq) and the optimization and conclusions are based on only one tissue (PBMCs). The utility of this study would be increased if both DOGMA-seq and TEA-seq were considered (1), the conclusions were drawn based on multiple tissues (2), and other well-established technologies for single-cell multi-omics were considered in the comparisons (e.g. 10x multiome for concurrent scRNA+scATAC) (3).

*R: We thank the reviewer for providing us with suggestions for improvement of our manuscript.*

*(1) We apologize for not pointing out more clearly that the published DOGMA-seq and TEA-seq protocols incorporate similar modifications to the 10x Genomics Multiome ATAC + Gene Expression protocol, with only a few differences. According to the TEA-seq paper[2], cells subjected to the TEA-seq assay were permeabilized using an isotonic, 0.01% digitonin (DIG)-containing buffer on ice for 5 minutes. This DIG cell permeabilization condition was one of two alternative cell permeabilization conditions described in the DOGMA-seq paper[3]. The DOGMA-seq developers also introduced a “low-loss lysis” (LLL) cell permeabilization condition. Thus, the major difference between DOGMA-seq and TEA-seq is that the DOGMA-seq protocol offers the option to use either the DIG or LLL methods for cell permeabilization, while the TEA-seq protocol uses the DIG method for cell permeabilization. Both the DIG and the LLL cell permeabilization conditions were considered in our manuscript.*

*(2) We agree that the scope of our study would have been considerably wider if we would have optimized and tested these novel single-cell multimodal omics technologies in multiple tissues. However, DOGMA-seq/TEA-seq are expensive, and since there is no universal protocol for different tissues, we chose to conduct a timely, independent optimization and validation study in PBMCs, one of the most widely used tissues in biomedical research. Based on feedback that we have already received from several colleagues, we feel that our in-depth, timely, and focused study will attract broad interest from investigators who are considering single-cell multimodal omics analysis to answer research questions in the fields of immunity and inflammation. We feel that optimizing these technologies for a wide range of tissues is beyond the scope of our Short Reports manuscript.*

*(3) A comprehensive comparison of single-cell trimodal omics measurements to the 10x Genomics Multiome ATAC + Gene Expression protocol was already included in the TEA-seq paper[2], where both unstained nuclei and unstained permeabilized cells were considered. The authors found that TEA-seq and unstained nuclei were quite similar in terms of gene detection rate. In comparisons of single cell ATAC-seq metrics, they found that TEA-seq performed comparably to the Multiome ATAC + Gene Expression protocol using permeabilized cells, with higher fraction of reads in peaks (FRIP) than purified nuclei used for the same assay.*

2. Some of the analyses presented in the manuscript lack rigor and raise doubts about the validity of the conclusions. Specifically:

2a. Comparing the number of clusters obtained with a clustering algorithm (in this case Louvain implemented in Seurat) using fixed parameters is by no means a metric of information content or quality. There are many aspects that affect the number of clusters obtained in Louvain clustering (or any other clustering algorithm) and are not necessarily related to the quality or discreteness of the data. For example, the number of cells, the density of the latent space, the scale and distribution of pairwise distances, etc. A more rigorous approach should be used here. For example, the authors could subsample reads (not UMIs) and cells so that the libraries have the same number of cells and UMIs. Then, they could then consolidate the subsampled libraries in a common latent space (e.g. using Harmony) and cluster this latent space to identify distinct cell populations. They could then perform differential expression analysis for each cell population and compare the number of differentially expressed genes obtained in each cell population by using only cells from one library at a time. Alternatively, they could use notions from information theory to quantify the amount of information content.

*R: We thank the reviewer for the suggestion. We agree with the reviewer that “comparing the number of clusters obtained with a clustering algorithm (in this case Louvain implemented in Seurat) using fixed parameters is by no means a metric of information content or quality”. However, we believe that the subsampling strategy suggested by the reviewer might not be the best choice to address concerns regarding performance of the clustering algorithm, information content or quality. All of our processed RNA library sequencing data (both CITE-seq and DOGMA-seq) had percent duplicates (a measure of sequencing saturation which is a function of library complexity and sequencing depth, according to 10x Genomics) higher than 76.7%. All of our processed ADT library sequencing data (both CITE-seq and DOGMA-seq) had percent duplicates higher than 61.1%. All of our processed ATAC library sequencing data had percent duplicates higher than 51.4%. These high percent duplicates indicate that we extracted a high proportion of the information available in our libraries, which may have variable complexity and quality between the methods used to generate them. We observed that unique reads could be filtered during subsampling of reads, resulting in less comparable percent duplicates between different permeabilization conditions or different assays. Furthermore, 10x Genomics cellranger-arc does not have a parameter to force the number of called cells, which makes it difficult to simultaneously subsample reads and cells.*

*Here, we propose an alternative strategy, as the reviewer suggested, to assess the clustering based on different modality or a weighted combination of them. We used the original libraries before subsampling to perform the analysis, noting that subsampling leads to the loss of information captured within each library. We no longer performed clustering using fixed parameters, and we did not interpret the number of clusters identified. As the reviewer suggested, we performed clustering with a series of resolution, ranging from 0.02 to 0.3, and we assessed the purity of identified clusters using a recently published entropy-based statistic, ROGUE[1]. We acknowledge that ROGUE had to be calculated based on RNA data, while our clustering was based on 3WNN/2WNN, RNA, ADT, and ATAC spaces.*

*For data generated using DOGMA-seq with DIG versus LLL cell permeabilization conditions, we separately performed clustering in their own 3WNN, RNA, ADT, and ATAC spaces. We found that DIG had a higher average ROGUE (higher purity of identified clusters) than LLL, given a similar resolution or number of clusters when clustering based on ADT and ATAC spaces (Figure 1). However, DIG and LLL had comparable average ROGUE, given similar resolution or number of clusters, when clustering based on 3WNN and RNA spaces. This supports our conclusion that we observed “higher quality ADT and ATAC libraries after DIG permeabilization”.*


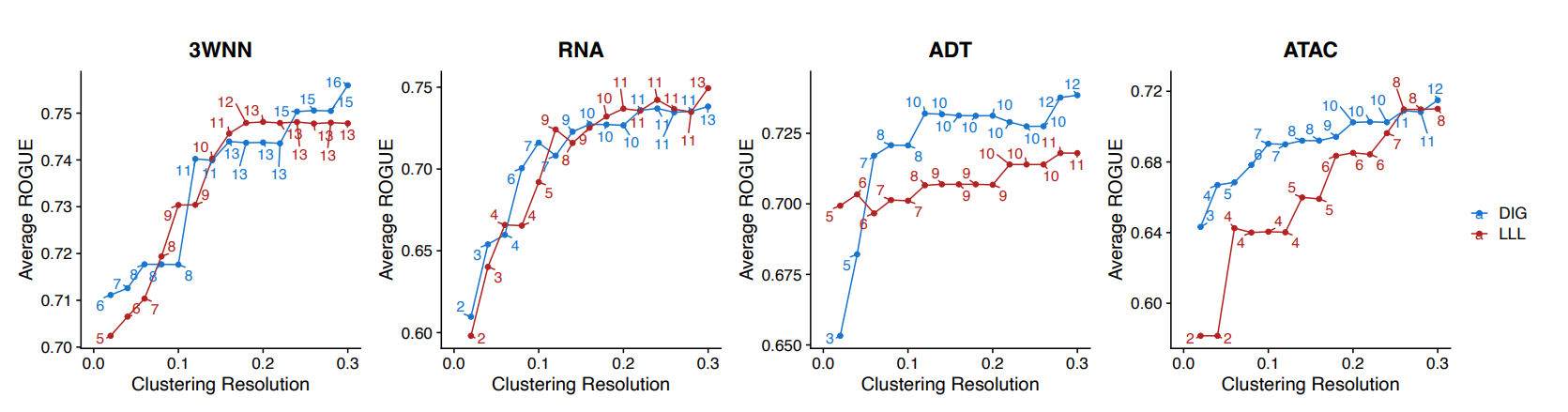


*Figure 1*

*Number of clusters identified under each resolution is labeled in the figure.*

*We also modified the comparison between CITE-seq and DOGMA-seq in the revised version of our manuscript. When we prepared sequencing libraries for the comparison between CITE-seq and DOGMA-seq, we loaded different aliquots of the pool of hashtagged cells from different samples into two different GEM chip wells for each assay (four wells in total for CITE-seq and DOGMA-seq). In the original version of our manuscript, we included cells from both GEM chip wells for each assay in our analysis, but we realized that the numbers of cells in our comparison between CITE-seq and DOGMA-seq would be more comparable to the comparison between the DIG and LLL conditions if only one GEM chip well for each assay was used (two wells in total for CITE-seq and DOGMA-seq). We now present results from analysis of only one GEM chip well per assay in the revised version of our manuscript.*

*For data generated using CITE-seq versus DOGMA-seq (RNA library of CITE-seq was aligned to exons only, while RNA library of DOGMA-seq was aligned to both exons and introns, as in our original manuscript), we separately performed clustering in their own 3WNN (DOGMA-seq)/2WNN (CITE-seq), RNA, and ADT spaces. We found that CITE-seq had higher average ROGUE than DOGMA-seq, given similar resolution or number of clusters, when clustering based on 3WNN/2WNN, RNA and ADT spaces (Figure 2). We now conclude that leveraging the additional ATAC library cannot fully compensate DOGMA-seq’s inferior quality in RNA and ADT libraries, compared with CITE-seq.*


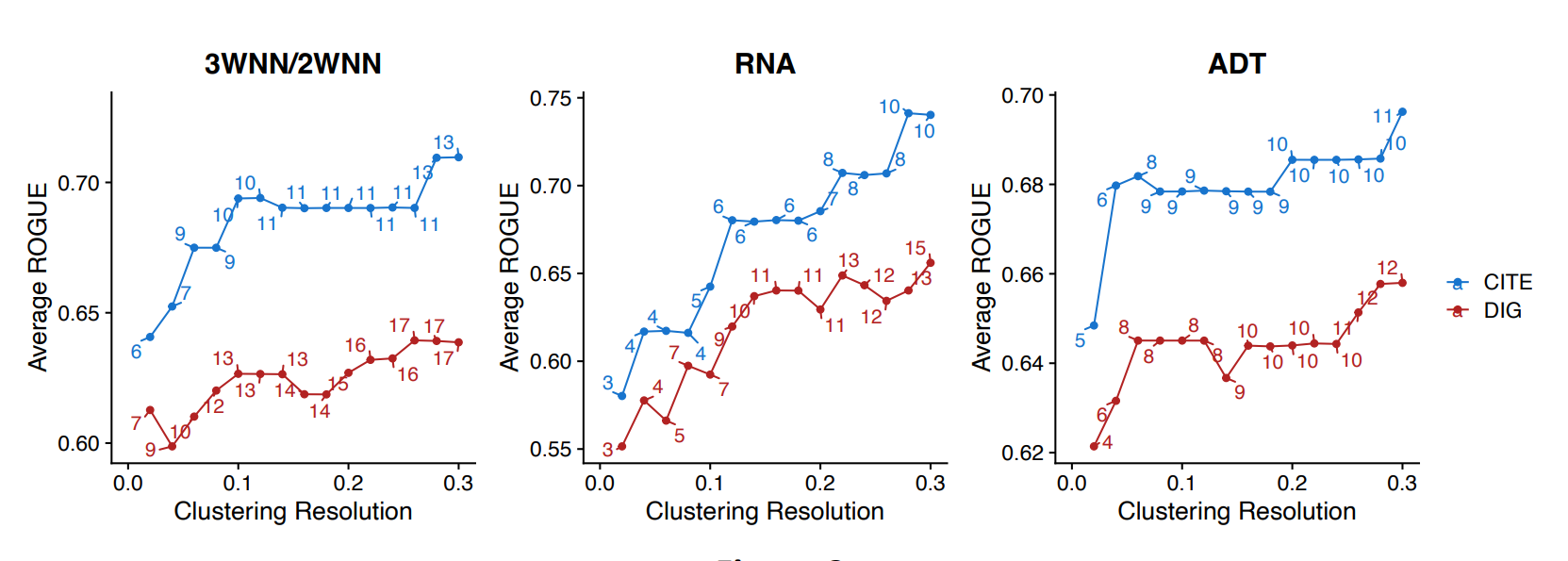


*Figure 2*

*Number of clusters identified under each resolution is labeled in the figure.*

*We also include a comparison between CITE-seq and DOGMA-seq, where RNA libraries were aligned to both exons and introns for CITE-seq and DOGMA-seq, like what was done in the TEA-seq paper. However, while we understand that this comparison may be fairer, we believe that it is also less practical, since single cell RNA-seq reads from intact cells (e.g., CITE-seq) are generally aligned to exons only. We found that DOGMA-seq had higher average ROGUE than CITE-seq, given similar resolution or number of clusters, when clustering based on 3WNN/2WNN, RNA and ADT spaces (Figure 3). This surprising difference could be because mapping CITE-seq’s RNA libraries to introns is inappropriate.*


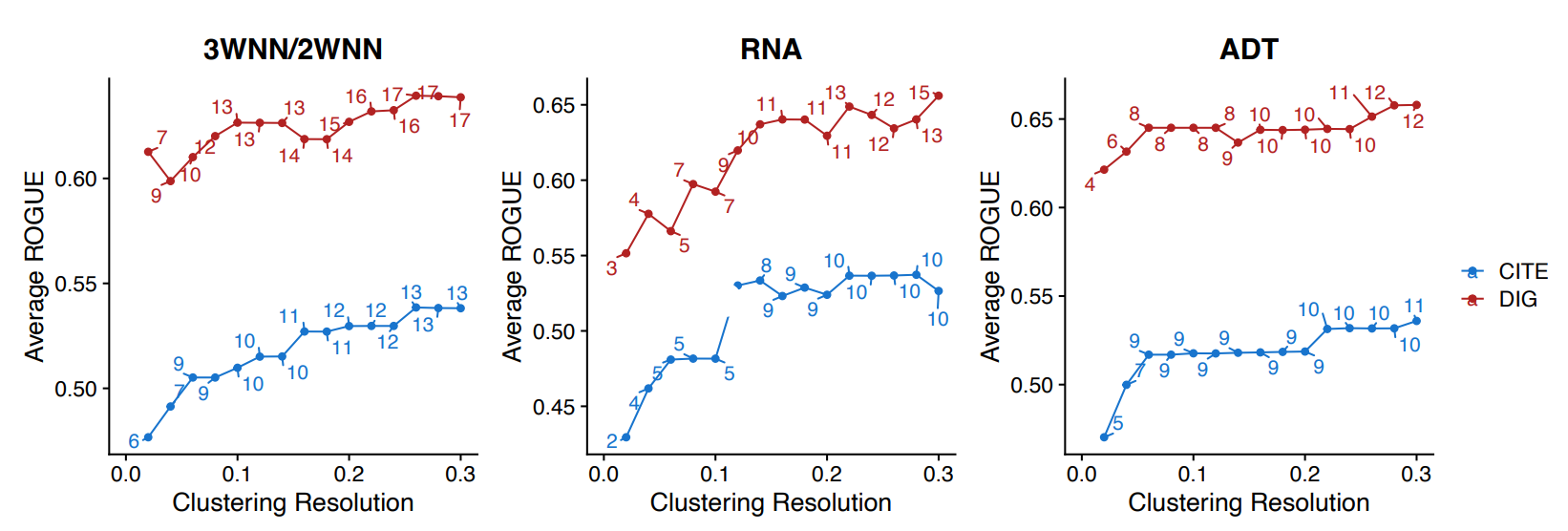


*Figure 3*

*Number of clusters identified under each resolution is labeled in the figure.*

*Hence, we include the more practical comparison between CITE-seq and DOGMA-seq in our revised manuscript main figures, but we are happy to change them to the comparison where CITE-seq and DOGMA-seq RNA libraries were aligned to both exons and introns if needed.*

2b. Comparing the transcriptome of CITE-seq and DOGMA-seq, when CITE-seq reads are aligned to exons and DOGMA-seq reads are aligned to both exons and introns does not make sense. Only comparisons where reads from both assays are aligned to the same features (Figs. S2D-F) should be considered.

*R: We thank the reviewer for the suggestion. As the reviewer noted, the suggested comparisons are shown in our Fig. S2D-F (aligned to both exons and introns, aligned to exons only, and aligned to introns only). However, as we stated above, while we understand that comparisons where RNA libraries were aligned to both exons and introns may be fairer, we believe that these comparisons are also less practical since single-cell RNA-seq reads from intact cells (e.g., CITE-seq) are generally aligned to exons only.*

*Similar to what was done in the TEA-seq paper, we show an analysis where CITE-seq and DOGMA-seq RNA libraries were aligned to both exons and introns, and we captured 13,206 and 14,172 cells for CITE-seq and DOGMA-seq, respectively, compared with 12,599 and 14,172 cells in our original analysis. With the consideration of both exon and intron reads in CITE-seq data, quality control metrics remained generally unchanged, except for detection of more genes (Figure 4).*


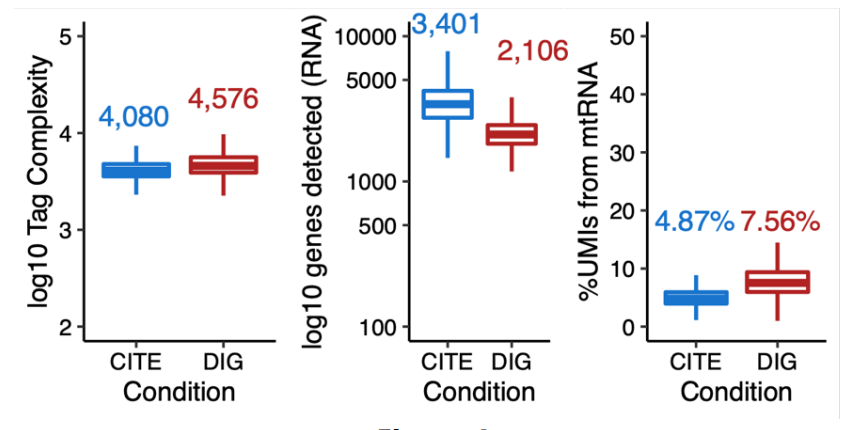


*Figure 4*

*As for correlation of log2FC, we observed results similar to those stated in the manuscript: “Gene fold changes after the addition of PGE2 to the cultures were still correlated at the pseudobulk level, but exon-dominated genes and intron-dominated genes formed two lines with different slopes” (Figure 5). As described in our manuscript, genes were dichotomized into exon-dominated genes and intron-dominated genes, according to proportion of exonic UMIs for each gene calculated based on DOGMA-seq data.*


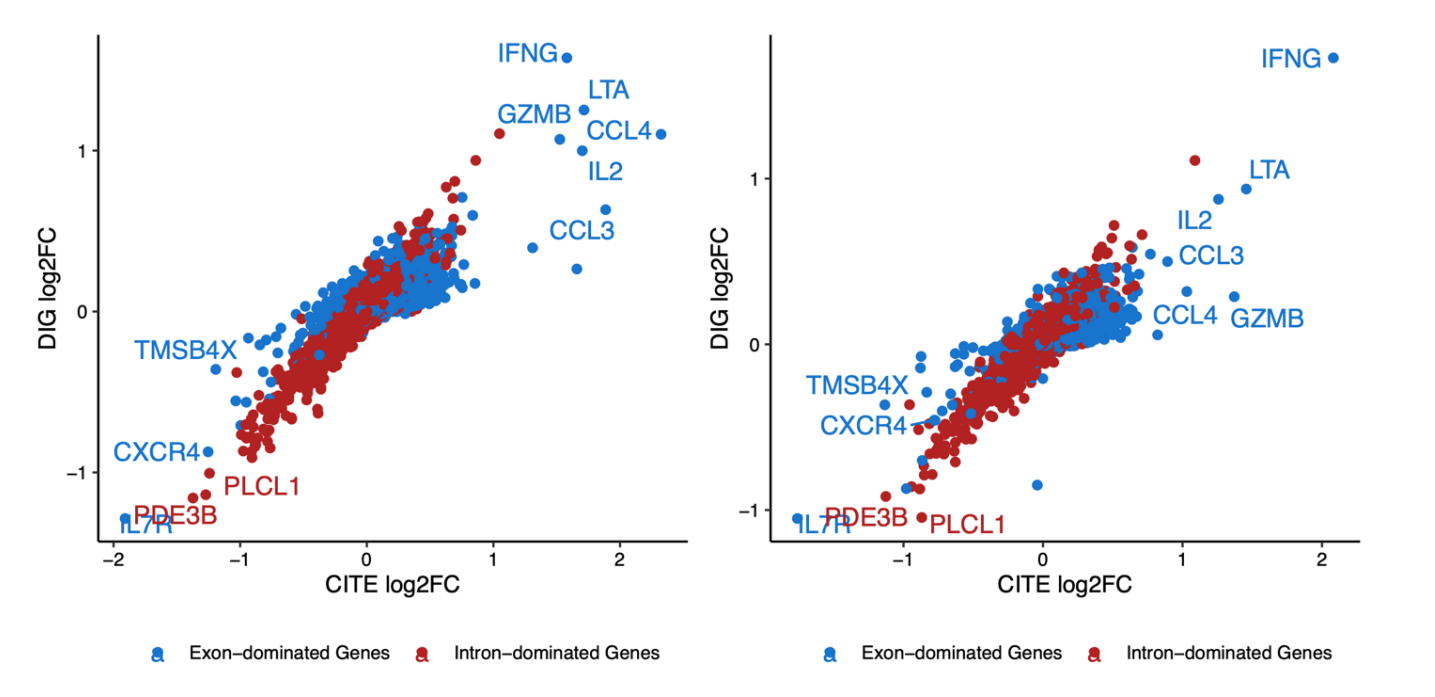


*Figure 5*

2c. A critical aspect that can negatively affect the quality of the libraries in DOGMA-seq is the presence of mRNA from lysed cells in the buffer. However, none of the metrics included in this study directly account for that. The study would greatly benefit from a human-mouse cell mixture experiment where the amount of background mRNA can be directly assessed in each permeabilization condition and technology by looking at the fraction of mouse UMIs when profiling human cells (and the fraction of human UMIs when profiling mouse cells).

*R: We thank the reviewer for raising this very important question. To address this question, we used DecontX, which accurately predicted contamination levels in a mouse-human mixture dataset[4], to estimate ambient RNA contamination rate for each cell under DIG and LLL cell permeabilization conditions. We filtered out all cells in filtered matrices (including singlets used in the analysis and doublets detected based on HTO) from raw matrices and used them to empirically estimate the distribution of ambient RNA. We found that DIG had a lower contamination rate than LLL (Figure 6), which is consistent with better preservation of the plasma membrane after DIG permeabilization. We also found that some CD4+ T cells had higher contamination rate than other cells. According to the DecontX paper[4], the median of contamination rate for singlets in a PBMC dataset (subjected to typical scRNA-seq) was 7.02%, with a range from 0.07% to 65.64%. We inferred that the median 16.7% observed under DIG condition was partially comparable and acceptable, given the nature of permeability. Besides, we* *hypothesize that the higher contamination rate could also partially explain the higher gene detection rates in LLL, where the additional detected genes could represent ambient RNA.*


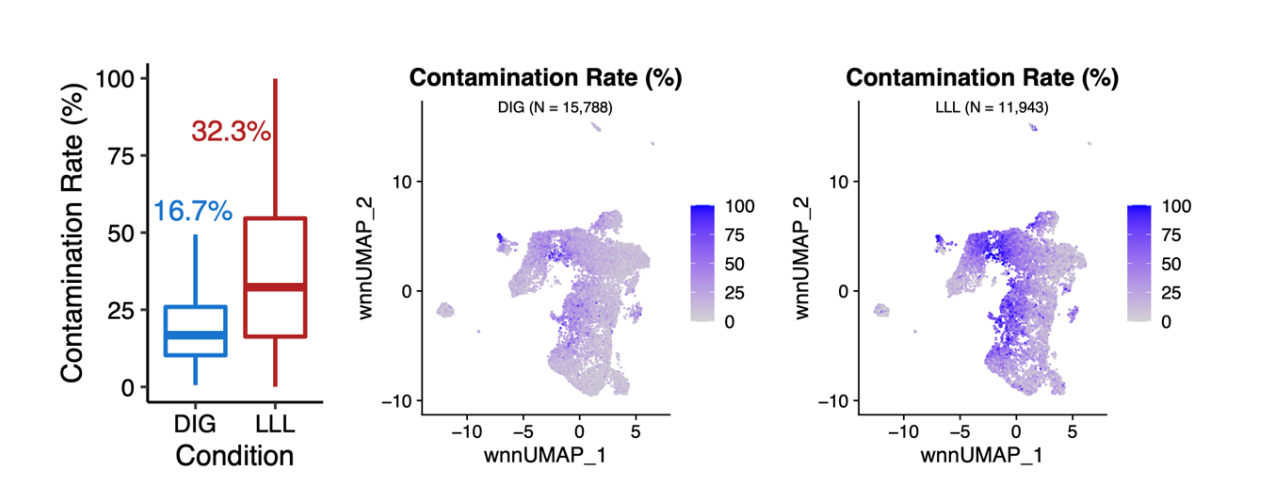


Figure 6

We tried to explore the differences in gene detection rates based on original RNA count matrices or decontaminated RNA count matrices (generated by DecontX). We found no difference if gene detection rates were defined as the fraction of cells with UMIs > 0 (Figure 7A, 7B). When we changed the definition to the fraction of cells with UMIs > 1, we found that many genes with higher detection rates in LLL have only one copy (Figure 7C), which is not common for synthesized RNA. The differences in gene detection rates were smaller when they were based on decontaminated RNA count matrices (Figure 7D), except for RPL/RPS genes. Based on these findings, we inferred that there is little difference between DIG and LLL in true gene detection rates for the majority of biologically meaningful genes, and that the higher gene detection rates in LLL are likely due to RPL/RPS genes and ambient RNA. This further supports our conclusion that DIG is the preferred cell permeabilization condition for the DOGMA-seq assay.


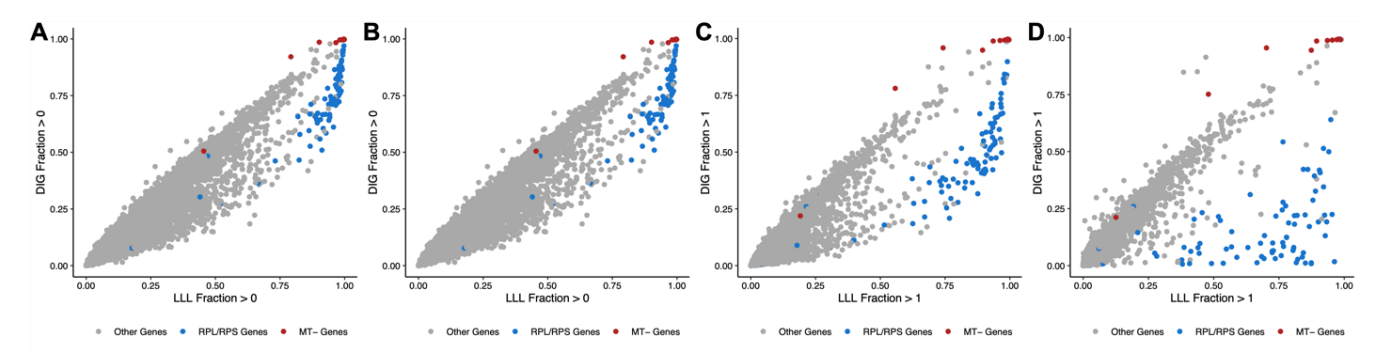


Figure 7

*(A) Gene detection rates, defined as the fraction of cells with UMIs > 0, were calculated based on original RNA count matrices.*

*(B) Gene detection rates, defined as the fraction of cells with UMIs > 0, were calculated based on decontaminated RNA count matrices.*

*(C) Gene detection rates, defined as the fraction of cells with UMIs > 1, were calculated based on original RNA count matrices.*

*(D) Gene detection rates, defined as the fraction of cells with UMIs > 1, were calculated based on decontaminated RNA count matrices.*

*To reduce the impact of the choice of background, we also merged the filtered raw matrices for DIG and LLL and used the merged matrix to estimate the distribution of ambient RNA, and the conclusion remained unchanged (Figure 8).*


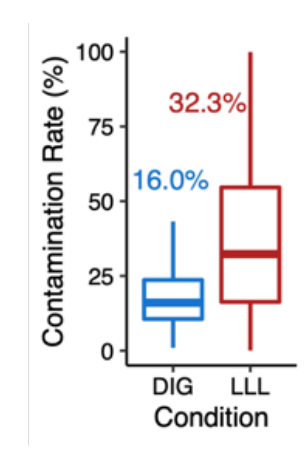


*Figure 8*

Reviewer #2

1. Line 48 - spelling of DOGMA

*R: We thank the reviewer for pointing out our typo.*

2. A reference/supplementary figure to the change in lysis time and concentration of DIG for the lysis step would be advantageous. The authors also state that after 1 min incubation in 0.0075% DIG 0.5% of the cells showed signs of permeabilization. Is this what the authors intended to state in the results section?

*R: We sincerely apologize for this typo and are very glad that the reviewer pointed out this important issue. We intended to state “99.5% of the cells treated with 0.0075% DIG showed red fluorescence indicative of propidium iodide entry into cells with compromised membranes, but lesser concentrations of DIG resulted in lower proportions of permeabilized cells.”*

3. The results section states that each cell population was then split into the DIG and LLL permeabilization groups, does this mean that all activation/stimulation steps were treated separated or combined together. It is not very clear from the results section.

*R: We apologize for the unclear description in the results section. We have made this clearer in the legend for Figure 1A. Each of four aliquots of T cells from a human donor were activated and stimulated under a different stimulation condition (total of four stimulation conditions in four tissue culture wells) in a 12-hour tissue culture. Each of two aliquots of cells from each of the four tissue culture wells were then labeled with a unique hashtag (total of eight unique hashtags). Approximately equal numbers of the eight uniquely hashtagged cell populations were then pooled and labeled with antibody cocktail. An aliquot of the hashtag- and antibody cocktail-labeled pool of cells was permeabilized with DIG, and another aliquot of the hashtag- and antibody cocktail-labeled pool of cells was permeabilized with LLL prior to the subsequent DOGMA-seq library preparation steps.*

4. How is tag complexity measured?

*R: Tag complexity estimates number of distinct tags for a cell, given the number of distinct tags observed for the cell and number of total tags observed for the cell.*

*Lander-Waterman equation that states:*

*C/X = 1 - exp(-N/X )*

*where*

*X = number of distinct molecules in library*

*N = number of read pairs*

*C = number of distinct fragments observed in read pairs*

*Tag complexity estimates X for a cell, given C and X for the cell.*

5. The authors state that whilst differential cell surface protein detection was seen between the two methods, this did not result in different protein tag signals. Could the authors then explain what was the differential measure that could be detected, was it different numbers of UMIs per ADT, dynamic range of signal?

*R: Protein tag detection rates assess to what extent a kind of tag is detectable in all the cells (the fraction of cells with UMIs > 0). Differential protein tag signals, which compare protein tag signals in cells activated and cultured under IL-1β and IL-23 vs cells activated and cultured under IL-1β, IL-23, and PGE2 stimulation conditions at the pseudo-bulk level, assess whether biological differences between the two stimulation conditions are detectable. One possible explanation is that most cells assayed using CITE-seq have UMIs > 0 for the majority of the 163 kinds of tags, but the differences in protein tags between the two stimulation conditions are detectable by both CITE-seq and DOGMA-seq, although the ranges of the UMI counts for the 163 kinds of tags could differ between CITE-seq and DOGMA-seq.*

6. The authors state that they can detect a slightly higher detection rate for some genes after LLL permeabilization (Fig 1L), is there any specific about these genes which have a higher detection level in the LLL procedure?

*R: The top 15 genes with a higher detection rate under LLL are shown below (Table 1). We found that a large proportion of these genes are ribosome and ubiquitin genes.*

*Table 1 Top 15 Genes with a Higher Detection Rate under LLL*

*Gene Detection Rate (DIG) Detection Rate (LLL) Type Proportion of Exonic UMIs (DIG) Proportion of Exonic UMIs (LLL) Difference in Detection Rate (LLL - DIG)*

*AL627171.2 0.184 0.633 Other Genes 1.000 1.000 0.449*

*ATP5F1E 0.419 0.858 Other Genes 0.969 0.992 0.440*

*UQCRB 0.222 0.652 Other Genes 0.993 0.995 0.429*

*NACA 0.459 0.870 Other Genes 0.953 0.988 0.411*

*RPL22 0.509 0.912 RPL/RPS Genes 0.916 0.969 0.403*

*TMA7 0.358 0.719 Other Genes 0.897 0.976 0.361*

*RPL4 0.466 0.824 RPL/RPS Genes 0.954 0.989 0.358*

*SRP14 0.276 0.628 Other Genes 0.977 0.993 0.353*

*UBB 0.304 0.657 Other Genes 0.997 0.997 0.353*

*SERF2 0.390 0.739 Other Genes 0.988 0.995 0.349*

*RPL35 0.558 0.905 RPL/RPS Genes 0.987 0.993 0.346*

*RPL12 0.611 0.957 RPL/RPS Genes 0.963 0.995 0.346*

*RPL24 0.585 0.928 RPL/RPS Genes 0.905 0.979 0.344*

*ATP5MD 0.149 0.491 Other Genes 0.784 0.953 0.342*

*RPS10 0.527 0.866 RPL/RPS Genes 1.000 1.000 0.340*

7. Does the detection of more RPL/RPS genes actually provide more information/definition to the multimodal analysis?

*R: We have not excluded RPL/RPS genes from our analyses because it is possible that these genes may have biological relevance. Unlike mitochondrial genes, removing RPL/PRS genes is not widely recommended in practice. In addition, we didn’t observe a cluster that has enriched RPL/RPS genes, so they should not affect the clustering or annotation. Users may choose to apply filters to these genes in downstream analysis.*

8. Could the authors explain why the data had to be batch corrected using Harmony before running the 3WNN? Where all activation/stimulation conditions pooled together for the analysis?

*R: We only corrected batch effects between permeabilization conditions (DIG vs LLL) and assays (CITE-seq vs DOGMA-seq) using Harmony, when combining datasets. The batch effects between permeabilization conditions (DIG vs LLL) and assays (CITE-seq vs DOGMA-seq) were evident due to different ranges of signals. Besides, correcting these batch effects before running 3WNN was also suggested and performed in the original DOGMA-seq paper.*

*We observed little batch effects between the four stimulation conditions or the different healthy donors, and we didn’t correct for them since cells cultured under the four stimulation conditions or collected from different healthy donors were labeled with hashtags and ADTs and sequenced together. When we analyzed a single dataset (e.g., data under DIG condition including four stimulation conditions), batch correction wasn’t performed.*

9. Whilst it is true that an additional cluster can be seen in the UMAP plots for RNA (LLL) and ADT (DIG) do these clusters actually improve the definition of the landscapes? Do the authors know the identity of these cells, are they the same sub-cell type, detected by the different omic methods?

*R: We now use an alternative strategy, which assessed the purity of identified clusters using an entropy-based statistic, ROGUE[1]. The results are shown in our response to Reviewer #1, 2a.*

10. The authors state that they can use the ATAC data to identify specific sub-types of T cells within the data. It would be better to state which markers/genes they are looking at in the text. Also would it not be of advantage to show the same gene/marker in all 3 omics to show the difference in detection, the authors have done this for CCR6 and RORC in two of the omics but not all three.

*R: We indicate which markers/genes we looked at to identify T cell subsets in the figures and/or the figure legends. We didn’t include RNA data for CCR4, CXCR3, and CCR5 because they were hardly detected in cells, and we were not able to interpret the RNA signals for those genes. The differences between the ADT and RNA data for those genes are good examples of the added value of ADT data for some genes (Figure 9).*


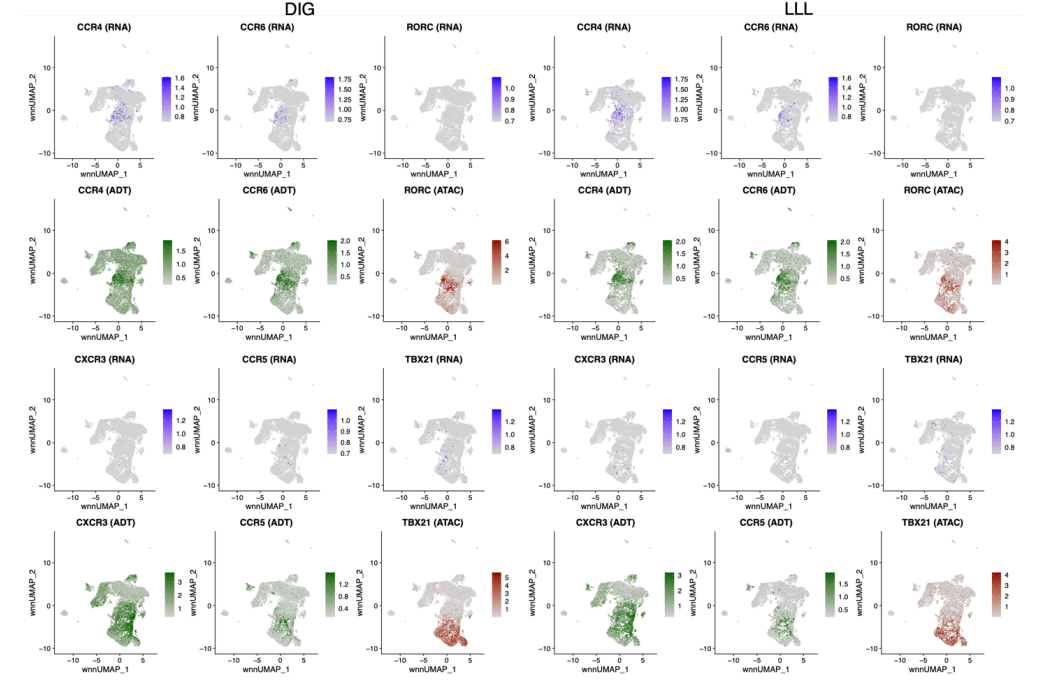


Figure 9

11. When showing the ATAC seq data on the wnnUMAP, what exactly are the authors showing, is this all the open regions detectable within the gene highlighted?

*R: The ATAC markers shown on the wnnUMAP plots were motif activity score, which is the deviations in chromatin accessibility across the set of regions (motifs)[5]. It was used to estimate gain or loss of accessibility within peaks sharing the same motif while controlling for technical biases.*

12. The authors do not conclusively prove or conclude at the end of Figure 1 which method is better to define the landscape. Do they benefit from having more information from the ADT, mRNA or ATAC to define the landscape? Is there really a qualitive or quantitative difference between the two methods?

*R: We apologize for the unclear conclusion. We observed that DIG has better quality ADT and ATAC libraries, and we inferred that the higher gene detection rates in LLL are likely due to RPL/RPS genes and ambient RNA (the results are shown in our response to Reviewer #1, 2c). Given the considerable* *difference in ADT and ATAC, as well as ambient RNA contamination, we believe that DIG is the better choice of permeabilization condition.*

13. It is not immediately clear from the text that the two donors were kept separately but used for both protocols, this should be made clearer in the schematic in Figure 2 or mentioned in the text.

*R: We apologize for the unclear description in the results section. We have made this clearer in the legend for Figure 2A. In our experiment that compared CITE-seq and DOGMA-seq with DIG cell permeabilization, each of four aliquots of T cells from two human donors were activated and stimulated under a different stimulation condition (total of four stimulation conditions in eight tissue culture wells) in a 12-hour tissue culture. The cells from each of the eight tissue culture wells were then labeled with a unique hashtag (total of eight unique hashtags). Approximately equal numbers of the eight uniquely hashtagged cell populations were then pooled and labeled with antibody cocktail. Different aliquots of the hashtag- and antibody cocktail-labeled pool of cells then underwent either CITE-seq or DOGMA-seq with DIG library preparation.*

14. Is there an explanation as to why the complexity of the ADT library is increased in the second DIG experiment? Is this degree of variability normal between repeats of the process and if this is the case then how can qualitative comparisons between the methods/technical repeats be made?

*R: We acknowledge there is a difference in the complexity of the ADT libraries between the two DIG experiments. We believe that they are not comparable, because the two DIG experiments were not head-to-head comparisons of cells from the same donor cultured at the same time and processed together to make sequencing libraries. Some variability between different experiments is not unexpected.*

15. The protein tag detection rates of DOGMA-seq are clearly lower than those of CITE-Seq and yet the complexity of the library is higher than that of the CITE-Seq experiment. Does this signify that the complexity of the ADT library is not a good metric to determine the "success" of the protocol?

*R: Protein tag detection rates and tag complexity assess the ADT libraries in two different perspectives. Tag complexity estimates the number of distinct tags for a cell but does not distinguish the 163 kinds of tags. Protein tag detection rates assess to what extent a kind of tag is detectable in all the cells (the fraction of cells with UMIs > 0). One possible explanation is that most cells assayed using CITE-seq have UMIs > 0 for the majority of the 163 kinds of tags (but with relatively low intensity), while the total number of unique tags binding to a cell was not as high as that for DOGMA-seq.*

16. The same markers highlighted in Fig 2Fi-ii are also shown in Fig 1, does this again show that these markers are so upregulated compared to any other surface protein, that they dominate the ADT library?

*R: In Fig 1K and Fig2Fi-ii, we show log2FC for protein tags (cells activated and cultured under IL-1β and IL-23 vs cells activated and cultured under IL-1β, IL-23, and PGE2 stimulation conditions) and the high correlation of log2FC between the two cell permeabilization conditions or the two different assays. We believe that the labeled markers reflect biological differences between the two stimulation conditions. The figures show that the biological differences were detectable regardless of which cell permeabilization condition (DIG or LLL) or assay (CITE-seq or DOGMA-seq) was used.*

17. What is the actual dynamic detection range of all the 163 protein tags in the ADT library when measured by the different protocols?

*R: We attach two pdf files (at the end of these responses) that can be used to compare the range of all 163 protein tags between different permeabilization conditions or assays. The expression level means log10(1 + protein tag count).*

18. Does this analysis suggest that whilst the number of genes detected using CiTE-Seq and DOGMA-Seq protocols is similar, the more robust expression is detected using the CITE-Seq protocol?

*R: We observed that CITE-seq had higher detection rates for almost all exon-dominated genes and some intron-dominated genes, whereas DOGMA-seq had higher detection rates for most intron-dominated genes (Fig. 2G). Our interpretation of this finding is that CITE-seq and DOGMA-seq have different strengths in gene detection rate.*

19. The authors claim that there are less clusters seen in the RNA and ADT UMAPs of the DOGMA-Seq experiments, due to better quality data in the CITE-Seq experiments. Supplementary figure 3 shows that there are more clusters in S3Aii and iii than in S3Bii and iii, so this statement is inaccurate. The ADT UMAP of the CITE-Seq experiment appears to be much better resolved than that of the DOGMA-Seq but the number of clusters is less in CITE-Seq when compared to DOGMA-Seq.

*R: We now use an alternative strategy, which assessed the purity of identified clusters using an entropy-based statistic, ROGUE[1]. The results are shown in the response to Reviewer #1, 2a.*

20. The predicted celltype labelling on all UMAPs is unsatisfactory, whilst it is understandable that the authors have corroborated the cell type association using Azimuth, the tri-modal approach taken in DOGMA-seq should allow much better celltype prediction as the authors elude to in the text. It would be of benefit to the readers of this article if the authors tried to define the celltypes based on the individual clusters rather than combining clusters and labelling large areas of the UMAPs as CD4/CD8 T cells, this is not particularly informative to the reader.

*R: We apologize that the predicted cell type labels lacked detail. Here we show predicted cell type labels with more details (Figure 10). We acknowledge that fine manual annotation for clusters exceeds our current working bandwidth, and we note that Azimuth was also used in the DOGMA-seq paper to annotate cell types [3].*


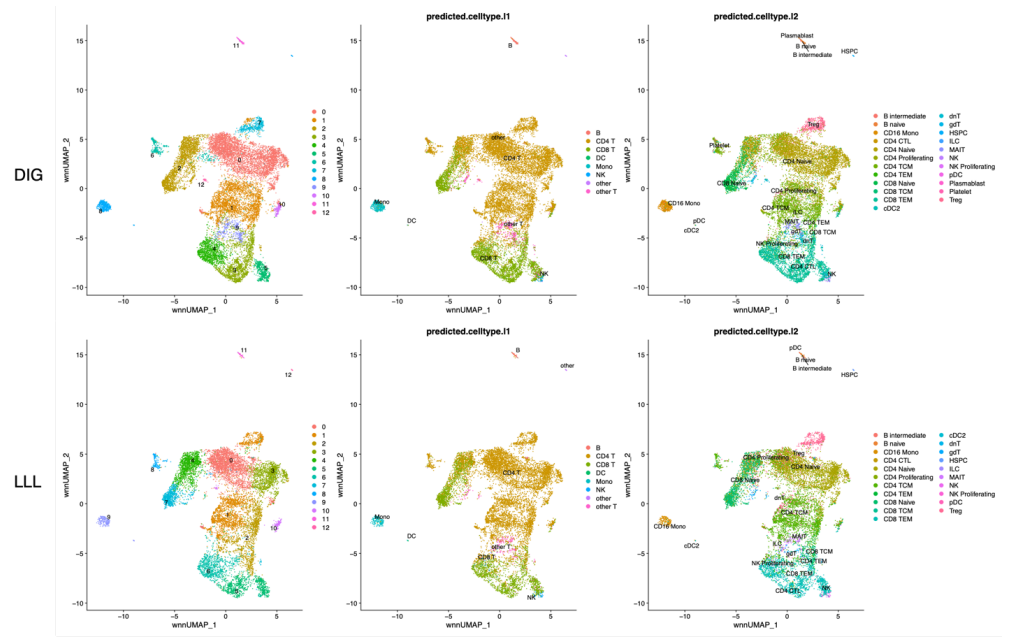


*Figure 10*

21. The authors have also performed several different stimulations of the isolated T cells, where is all of this data from the individual stimulations? It would be of benefit to the general audience of this manuscript to be able to see how these stimulations change the landscape, whilst it is beyond the scope of this manuscript to delve into the differences seen in the epigenetic landscape upon stimulation and how this correlates with gene expression changes or surface protein phenotype it would be reassuring to know that this data was available as a resource for the community.

*R: We merged cells cultured under four stimulation conditions to form a single dataset for each group of experiments but used some of the conditions to assess whether biological differences at the RNA and ADT levels are detectable. We feel that additional differential expression analyses across the four stimulation conditions are beyond the scope of this manuscript focusing on comparisons of different permeabilization conditions and different assays. In fact, the work reported in this manuscript was partly motivated by our desire to select the best single cell multimodal omics method for differential analysis in many more samples going forward.*

22. In the methods section there is mention of using Hastags to separate the donors and condition, this was not mentioned within the manuscript, does this additional layer of multiplexing affect the experimental procedure?

*R: Please see response to Reviewer #2, 3. above. Our use of cell hashing to uniquely label cells from each human subject donor x cell culture condition before the cells were pooled for ADT labeling and other downstream library prep processes mitigates potential batch effects that might have been observed if the cells from each human subject donor x cell culture condition would have been processed separately. Cell hashing also enables more cost-effective single cell sequencing library prep since hashtagged, pooled cells from unique human donor x cell culture conditions can be processed together in a 10x Genomics library prep chip lane rather than in separate library prep chip lanes that would have been necessary without cell hashing. One of the advantages of DOGMA-seq compared to the 10x Genomics Multiome ATAC + Gene Expression protocol is that DOGMA-seq enables cell hashing.*

23. The authors mention that the raw and processed data will be deposited to GEO upon acceptance of the paper, the data needs to be submitted (but kept private) before the paper can be accepted and the same for the analysis code.

*R: We have deposited the raw and processed data into GEO (GSE200417, the reviewer could access the data with the following secure token: wbqjwqwmnleptgt) and will make it available to the public upon acceptance of the paper. We believe this valuable trimodal single-cell dataset can serve as a unique reference to the field for bioinformatics method developments.*

*Bibliography*

*1. Liu B, Li C, Li Z, Wang D, Ren X, Zhang Z. An entropy-based metric for assessing the purity of single cell populations. Nat Commun. 2020;11:3155. doi:10.1038/s41467-020-16904-3.*

*2. Swanson E, Lord C, Reading J, Heubeck AT, Genge PC, Thomson Z, et al. Simultaneous trimodal single-cell measurement of transcripts, epitopes, and chromatin accessibility using TEA-seq. Elife. 2021;10. doi:10.7554/eLife.63632.*

*3. Mimitou EP, Lareau CA, Chen KY, Zorzetto-Fernandes AL, Hao Y, Takeshima Y, et al. Scalable, multimodal profiling of chromatin accessibility, gene expression and protein levels in single cells. Nat Biotechnol. 2021;39:1246–58. doi:10.1038/s41587-021-00927-2.*

*4. Yang S, Corbett SE, Koga Y, Wang Z, Johnson WE, Yajima M, et al. Decontamination of ambient RNA in single-cell RNA-seq with DecontX. Genome Biol. 2020;21:57. doi:10.1186/s13059-020-1950-6.*

*5. Schep AN, Wu B, Buenrostro JD, Greenleaf WJ. chromVAR: inferring transcription-factor-associated accessibility from single-cell epigenomic data. Nat Methods. 2017;14:975–8. doi:10.1038/nmeth.4401.*

**Second round of review**

**Reviewer 1**

I would like to thank the authors for their clarifications. In the revised the manuscript they have partially addressed the concerns about the original version of the manuscript. In particular:

1. The scope of the study is too narrow and will likely be of interest to a reduced number of people.

The response of the authors adds clarity about their viewpoint. However, the scope of the study has not changed, and similar comparisons between LLL and DIG and between DOGMA-seq and CITE-seq were already done in the original publication of DOGMA-seq. If the editor thinks the scope is acceptable, my suggestion would be that the authors try to more clearly emphasize what is new in this work with respect to the DOGMA-seq paper.


2a. Comparing the number of clusters obtained with a clustering algorithm (in this case Louvain implemented in Seurat) using fixed parameters is by no means a metric of information content or quality.

In the revised manuscript, the authors provide a more rigorous approach to evaluate the information content. They perform clustering using different values of the resolution parameter in Seurat and evaluate the transcriptomic variability within each cluster by using the entropy-based statistic ROGUE (Liu et al. Nat. Communications 11, (2020)). This approach overcomes the limitations of the approach used in the original version of the manuscript.


2b. Comparing the transcriptome of CITE-seq and DOGMA-seq, when CITE-seq reads are aligned to exons and DOGMA-seq reads are aligned to both exons and introns does not make sense.

I continue to disagree with the authors about this point. I am sorry for being insistent but comparing single-cell RNA-seq datasets that have been mapped to a different set of genomic features is not a valid comparison for evaluating two RNA-seq libraries. Both CITE-seq and DOGMA-seq use the same poly-T-priming 3’-biased approach for generating their RNA-seq library. The authors argue that the RNA-seq library of CITE-seq is usually aligned to only exons. This is not accurate. 3’-biased single-cell RNA-seq libraries in general (including those of CITE-seq) are often aligned to exons+introns (e.g. for RNA-velocity) or to just exons. All comparisons need to be done between RNA-seq libraries that have been aligned to the same set of genomic features (either exons, or exons+introns).


2c. A critical aspect that can negatively affect the quality of the libraries in DOGMA-seq is the presence of mRNA from lysed cells in the buffer.

The authors have fully addressed this point by using DecontX (Yang et al., Genome Biol. 21 (2020)) to estimate the amount of background RNA in each method.


Minor point: Significance needs to be quantified statistically in Figs. 1b-h, 2b-d, S1g and S2a-c.

**Reviewer 2**

The authors have satisfactorily responded to all of my queries regarding the manuscript.
